# Diversity of metallo-β-lactamase-encoding genes found in distinct species of *Acinetobacter* isolated from the Brazilian Amazon Region

**DOI:** 10.1590/0074-02760190020

**Published:** 2019-05-30

**Authors:** Danielle Brasiliense, Rodrigo Cayô, Ana Paula Streling, Carolina S Nodari, Rafael R Barata, Poliana S Lemos, Janaina M Massafra, Yan Correa, Igor Magalhães, Ana C Gales, Roberta Sodré

**Affiliations:** 1 Instituto Evandro Chagas, Seção de Bacteriologia e Micologia, Ananindeua, PA, Brasil; 2 Universidade Federal de São Paulo, Instituto de Ciências Ambientais, Químicas e Farmacêuticas, Departamento de Ciências Biológicas, Setor de Biologia Molecular, Microbiologia e Imunologia, Diadema, SP, Brasil; 3 Universidade Federal de São Paulo, Departamento de Medicina, Escola Paulista de Medicina, Laboratório Alerta, Disciplina de Infectologia, São Paulo, SP, Brasil; 4 Instituto Evandro Chagas, Centro de Inovação Tecnológica, Ananindeua, PA, Brasil; 5 Hospital Fundação Santa Casa de Misericórdia do Pará, Belém, PA, Brasil

**Keywords:** metallo-β-lactamase, non-*baumannii Acinetobacter* species, polymyxin resistance, nosocomial infection, ICU, Tn*125*

## Abstract

**BACKGROUND:**

The multidrug resistance (MDR) phenotype is frequently observed in *Acinetobacter baumannii*, the most clinically relevant pathogenic species of its genus; recently, other species belonging to the *A. calcoaceticus-A. baumannii* complex have emerged as important MDR nosocomial pathogens.

**OBJECTIVES:**

The present study aimed to verify the occurrence of metallo-β-lactamase genes among distinct *Acinetobacter* species in a hospital located in the Brazilian Amazon Region.

**METHODS:**

Antimicrobial susceptibility profiles were determined by broth microdilution. The genetic relationships among these isolates were assessed by pulsed-field gel electrophoresis (PFGE) and multilocus sequence typing (MLST). Pyrosequencing reads of plasmids carrying the *bla*_NDM-1_ gene were generated using the Ion Torrent™ platform sequencing.

**FINDINGS:**

A total of six isolates carried *bla*_NDM-1_: *A. baumannii* (n = 2), *A. nosocomialis* (n = 3), and *A. pittii* (n = 1); three carried *bla*_IMP-1_: *A. baumannii*, *A. nosocomialis*, and *A. bereziniae*. Resistance to colistin was observed for an NDM-1-producing *A. nosocomialis* isolate. Diverse PFGE patterns and sequence types were found among *A. nosocomialis* and *A. baumannii* isolates. The *bla*_NDM-1_ sequence was inserted in a Tn*125* transposon, while the *bla*_IMP-1_ was found as a gene cassette of the class 1 integron In*86*.

**MAIN CONCLUSIONS:**

To the best of our knowledge, this is the first report describing the dissemination of *bla*_NDM-1_ among distinct *Acinetobacter* species recovered from the same hospital in South America.

The World Health Organization (WHO) has recognised carbapenem-resistant *Acinetobacter baumannii* as a critical priority pathogen for antimicrobial development and research.^1^ The multidrug resistance (MDR) phenotype is frequently observed in *A. baumannii*, which is the most clinically relevant species of its genus,^2^ and recently, other species belonging to the *A. calcoaceticus-A. baumannii* complex have emerged as important MDR nosocomial pathogens.^3^ ^,^ ^4^ ^,^ ^5^ ^,^ ^6^

According to the most recent report from the Brazilian Health Surveillance Agency (ANVISA), *Acinetobacter* spp. were ranked as the fourth most frequent pathogen (n = 2,129; 12.6%) causing catheter-related bloodstream infections (CR-BSI) in Brazilian intensive care units (ICUs).^7^ Eighty-five percent of *Acinetobacter* spp. reported to ANVISA was resistant to carbapenems. Therefore, polymyxins have constituted the first therapeutic option for the treatment of serious carbapenem-resistant *Acinetobacter* spp. infections. In Brazil, carbapenem resistance among *A. baumannii* clinical isolates is mainly associated with the production of the class D carbapenemase (CHDL) variants OXA-23, OXA-72, and OXA-143 at a lower frequency.^3^ ^,^ ^8^ ^,^ ^9^ The spread of high-risk clones of producers of OXA-23 or OXA-72 explain the high carbapenem resistance rates observed among *Acinetobacter* spp. recovered from adults diagnosed with CR-BSI in Brazilian ICUs.^7^

In contrast, carbapenem resistance due to the production of metallo-β-lactamases (MβLs) has rarely been reported in *A. baumannii* as well as in non-*baumannii* species.^4^ ^,^ ^10^ ^,^ ^11^ IMP-type MβLs have only been reported in *Acinetobacter* spp. isolated from hospitals located in the state of São Paulo,^4^ ^,^ ^10^ south-eastern Brazil, while NDM-1-producing isolates have been sporadically described in hospitals located in the three different states of the Southern region.^5^ ^,^ ^6^ ^,^ ^11^

Herein, we report the spread of NDM-1 and IMP-1-producing *Acinetobacter* spp. causing complicated nosocomial infections at a Brazilian tertiary hospital located in the Brazilian Amazon Region (Northern Brazilian Region), and the emergence of polymyxin resistance in this group of pathogens.

## MATERIALS AND METHODS

*Bacterial isolates and phenotypic detection of MβL production* - A total of 478 non-duplicated *Acinetobacter* spp. clinical isolates were obtained between January 2012 and June 2016 at a tertiary teaching hospital with 408 beds located in the city of Belém, state of Pará, Brazil. The isolates were sent to Evandro Chagas Institute (IEC) for further characterisation. Among those isolates, nine (1.9%) showed reduced susceptibility to carbapenems and were positive by imipenem/imipenem+EDTA (0.5 M) double disk synergy test. Subsequently, the MβL phenotype was confirmed using imipenem/imipenem+EDTA Etest^®^ strips (bioMérieux, Solna, Sweden) according to the manufacturer’s instructions.

*Species identification and antimicrobial susceptibility testing* - Species identification was performed by partial sequencing of the *rpoB* gene, as previously described.^12^ Minimum inhibitory concentrations (MICs) of ampicillin/sulbactam, ceftazidime, cefotaxime, imipenem, meropenem, ciprofloxacin, amikacin, gentamicin, tobramycin, minocycline, tigecycline, polymyxin B, and colistin (Sigma-Aldrich, St. Louis, USA) were determined by cation-adjusted broth microdilution (Oxoid, Basingstoke, UK) following the European Committee on Antimicrobial Susceptibility Testing (EUCAST) recommendations. The susceptibility results were interpreted according to the current EUCAST breakpoints (http://www.eucast.org/clinical_breakpoints).

*Detection of carbapenemase-encoding genes* - Polymerase chain reaction (PCR) followed by DNA sequencing was performed for the detection of acquired CHDL (*bla*_OXA-23_-like, *bla*_OXA-24/40_-like, *bla*_OXA-58_-like, *bla*_OXA-143_-like) and MβL-encoding genes (*bla*_NDM_-like, *bla*_IMP_-like, *bla*_VIM_-like, *bla*_SIM_-like and *bla*_SPM_-like) using specific primers, as previously described.^8^ ^,^ ^9^ ^,^ ^10^ Amplicons were purified using the QIAquick Gel Extraction Kit (Qiagen, Courtaboeuf, France) according to the manufacturer’s instructions, and sequencing reactions were prepared using the BigDye Terminator Cycle Sequencing Kit (Applied Biosystems, Foster City, USA) and the ABI 3500 Genetic Analyser (Applied Biosystems, Perkin Elmer, USA). The nucleotide sequences and the derived protein sequences were analysed using the Lasergene Software Package (DNASTAR, Madison, USA), and then compared with those deposited in the GenBank database.

*Characterisation of plasmids and genetic context of MβL-encoding genes* - Genomic and plasmid DNA were extracted using the Wizard Genomic DNA purification kit (Promega, Madison, WI, USA) based on the manufacturer’s recommendations. The genetic structures surrounding the MβL-encoding genes were evaluated by PCR mapping followed by DNA sequencing using specific primers based on previously reported sequences.^4^ ^,^ ^10^ The plasmids carrying *bla*_NDM-1_ were digested with endonuclease S1 and resolved by pulsed-field gel electrophoresis (PFGE), followed by DNA-DNA hybridisation assay that was assessed by Southern blotting using a Hybond™-N^+^ nylon transfer membrane (GE Healthcare, Little Chalfont, UK).^10^ DIG-labelling of a *bla*_NDM-1_-specific probe and signal detection was performed using the DIG DNA Labelling and Detection Kit (Roche Diagnostics GmbH, Penzberg, Germany).

*Genotyping by PFGE and multilocus sequence typing (MLST)* - The clonal relationships of MβL-producing *Acinetobacter* spp. isolates belonging to the same species were investigated by PFGE using the CHEF DR II system (Bio-Rad, Hercules, California) and ApaI restriction enzyme (New England BioLabs, MA, USA), as previously described.^9^ MLST was performed for those isolates belonging to the *A. calcoaceticus-A. baumannii* complex by double-stranded DNA sequencing of internal regions of seven housekeeping genes (*cpn60*, *fusA*, *gltA*, *pyrG*, *recA*, *rplB*, and *rpoB*) according to the Institute Pasteur scheme. Determination of the sequence type (ST) was performed through the *A. baumannii* MLST website (http://pubmlst.org/abaumannii/). The relationships between novel and existing STs were surveyed using the eBURST program (http://eburst.mlst.net/).

*Genomic DNA sequencing, assembly, and plasmid sequence analysis* - Genomic DNA from representatives of each *Acinetobacter* species carrying *bla*_NDM-1_ was extracted and purified using the Wizard Genomic DNA purification kit (Promega, Madison, WI, USA) according to the manufacturer’s protocol. Single-end pyrosequencing reads of plasmids were generated using the Ion Torrent™ Personal Genome Machine™ (PGM) platform sequencing (Thermo Fisher Scientific, Carlsbad, CA). Raw sequence reads were trimmed and assembled *de novo* using plasmidSPAdes version 3.9.0 (http://bioinf.spbau.ru/spades) and MIRA v.1.0.4 software, respectively, followed by gap filling by manual assembly. Each draft plasmid was annotated by Rapid Annotations using Subsystems Technology (RAST) (http://rast.nmpdr.org) and further manually curated by Geneious v.9.1.6 (Biomatters Limited, Auckland, New Zealand) using Basic Local Alignment Search Tool (BLAST) against the non-redundant NCBI database (http://blast.ncbi.nlm.nih.gov/Blast.cgi).

*Nucleotide sequencing accession numbers* - The complete nucleotide sequences of plasmids pIEC383, pIEC37710, and pIEC38057 were submitted to GenBank under accession numbers MK053932, MK053933, and MK053934, respectively.

*Ethical approval* - Ethical approval for the study was obtained from Research Ethics Committee from Evandro Chagas Institute (process number: 655.019/CAAE: 24147014.8.0000.0019).

## RESULTS AND DISCUSSION

Among the nine carbapenem-resistant *Acinetobacter* spp. isolates phenotypically identified as MβL producers, six harboured *bla*_NDM-1_ (n = 6) and three carried *bla*_IMP-1_ (n = 3) genes (Table). No acquired CHDL-encoding gene was found in these isolates. The NDM-1-producing *Acinetobacter* spp. isolates were identified as *A. baumannii* (n = 2), *A. nosocomialis* (n = 3), and *A. pittii* (n = 1), while the three IMP-1-producing isolates were identified as *A. baumannii*, *A. nosocomialis*, and *A. bereziniae*. MβL-producing *Acinetobacter* spp. isolates were mostly recovered between March and August 2014 (n = 6). Most patients infected by these pathogens (n = 6; 66.7%) were hospitalised at paediatric or neonatal ICUs, as shown in Table; these results concur with those reported by previous studies. *Acinetobacter* spp. have previously been reported to be the 6th and 10th most frequent pathogens in Brazilian paediatric (n = 135; 6.5%) and neonatal ICUs (n = 199; 3.1%) in 2016, showing carbapenem resistance rates of 21% and 44%, respectively.^7^ While primary BSI was documented in five of six patients infected with NDM-1-producing *Acinetobacter* spp., the IMP-1-producing isolates were recovered from surgical wounds, peritoneal fluid, and urine. Only two patients infected with NDM-1-producing isolates died during hospitalisation (Table).

**TABLE:** Species identification, clinical data, genetic similarity, carbapenemase content, and antimicrobial susceptibility profile of nine carbapenem-resistant *Acinetobacter* spp. isolates

Isolate	Species	Date of isolation (mm/dd/yy)	Age	Underlying disease	Clinical specimen	Hospital unit	Outcome	MβL	PFGE	MLST	MIC (µg/mLl)												
											SAM	CRO	CAZ	FEP	IMP	MEM	CIP	AMK	GEN	TOB	MIN	TGC	COL
IEC304	*A. baumannii*	03/28/2014	66 y	Gastric cancer	Blood	General ward	Died	NDM-1	I	ST216/CC216	>256/4	>512	>256	>256	256	256	1	16	≤0.5	1	≤0.25	0.25	0.5
IEC383	*A. baumannii*	06/26/2014	NI	NI	Blood	Neonatal ICU	NI	NDM-1	II	ST464/CC464	>256/4	>512	>256	>256	256	256	0.5	4	≤0.5	0.5	≤0.25	0.25	0.5
IEC429	*A. baumannii*	08/15/2014	7 m	Neurologic disease	Urine	Neonatal ICU	Improved	IMP-1	III	ST464/CC464	>256/4	>512	>256	>256	128	256	64	32	1	2	≤0.25	0.5	≤0.25
IEC336	*A. nosocomialis*	05/08/2014	21 y	Postcesarean wound infection	Surgical wound secretion	Obstetric ward	Improved	NDM-1	1	ST433/CC782	>256/4	>512	>256	>256	256	>256	0.25	16	≤0.5	1	≤0.25	0.5	1
IEC343	*A. nosocomialis*	05/10/2014	6 m	Neurologic disease	Blood	Pediatric ICU	Died	NDM-1	2	ST1075/CC410	>256/4	>512	>256	256	128	128	0.25	4	≤0.5	1	≤0.25	0.25	1
IEC38057	*A. nosocomialis*	10/15/2016	6 m	Neurologic disease	Blood	Pediatric ICU	Improved	NDM-1	3	ST71/CC410	>256/4	>512	>256	>256	128	256	0.5	8	≤0.5	1	≤0.25	0.25	2
IEC195	*A. nosocomialis*	08/28/2013	1 m	Meningomyelocele	Surgical wound secretion	Neonatal ICU	Improved	IMP-1	4	ST279	>256/4	>512	256	256	128	256	0.5	32	16	8	≤0.25	2	64
IEC37710	*A. pittii*	10/12/2016	2 y	Neurologic disease	Blood	Pediatric ward	Improved	NDM-1	-	ST63/CC63	>256/4	>512	>256	>256	256	>256	0.25	2	≤0.5	0.5	≤0.25	0.25	≤0.25
IEC403	*A. bereziniae*	07/21/2014	7 y	Peritonitis	Peritoneal fluid	Neonatal ICU	Improved	IMP-1	-	-	64/4	>512	>256	256	128	256	32	32	8	8	≤0.25	0.5	2

AMK: amikacin; CAZ: ceftazidime; CIP: ciprofloxacin; COL: colistin; CRO: ceftriaxone; FEP: cefepime; GEN: gentamicin; ICU: intensive care unit; IMP: imipenem; m: months; MEM: meropenem; MIC: minimal inhibitory concentration; MIN: minocycline; NI: not informed; SAM: ampicillin/sulbactam; TGC: tigecycline; TOB: tobramycin; y: years.

Expectedly, MβL-producing *Acinetobacter* spp. isolates showed high MIC values to all β-lactams tested (Table). Contrastingly, minocycline and tigecycline were the most active antimicrobial agents against the isolates *in vitro*, with MIC_90_ values of ≤ 0.25 μg/mL and 0.5 μg/mL, respectively. Such results corroborated previous data that also showed good *in vitro* activity of minocycline against IMP-1- and IMP-10-producing *Acinetobacter* spp. isolates in Brazil.^4^ ^,^ ^10^ Despite its potential activity, the minocycline formulation for intravenous administration (Minocin^®^) is not yet available in Brazil. The NDM-1-producing *Acinetobacter* spp. isolates were also susceptible to ciprofloxacin and aminoglycosides. In contrast, most of the IMP-1-producing *Acinetobacter* spp. isolates were resistant to these drugs (Table). Interestingly, an IMP-1-producing *A. nosocomialis* isolate (IEC195) showed a high resistance rate to colistin (MIC, > 64 μg/mL). To the best of our knowledge, this is the first report of a colistin-resistant *A. nosocomialis* strain carrying *bla*_NDM-1_. Notably, a worrisome decrease in the susceptibility to polymyxins was observed among all NDM-1-producing *A. nosocomialis* and IMP-1-producing *A. bereziniae* isolates (MICs varying from 1-2 μg/mL). According to a study conducted by Vila-Farrés and colleagues, who evaluated induced colistin-resistant *A. nosocomialis* mutants, the loss of LPS due to mutations in the *lpxD* gene was found to be the main mechanism associated with this resistance phenotype in this species.^13^ In another study, Wang and colleagues reported high resistance rates to colistin (21.4%; n = 58/271) among *A. nosocomialis* isolates recovered from BSI in a hospital located in Taiwan during an 11-year period.^14^ The authors also reported that the colistin resistance could not be transferred by a conjugation assay,^14^ indicating a chromosomal mechanism that corroborated those findings previously reported by Vila-Farrés and colleagues.^13^ In addition, all 58 colistin-resistant *A. nosocomialis* isolates evaluated by Wang and colleagues were susceptible to ciprofloxacin,^14^ which was also observed in the colistin-resistant IEC195 strain (MIC 0.5 μg/mL) in the present study.

**Fig. 1:**
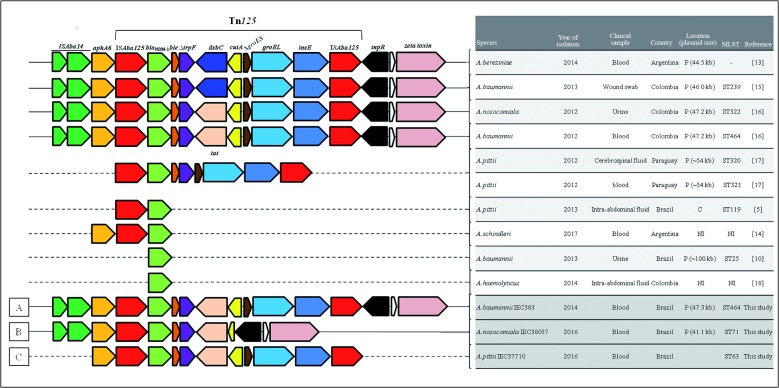
comparative analysis of Tn*125* backbone described in all current NDM-1-producing *Acinetobacter* species reported in South America with those found in the present study (A-C). Dashed lines represent the inner or surrounding regions of Tn*125* that were not sequenced. White arrows represent open reading frames (ORFs) that encoded hypothetical proteins. Genes and their transcription orientation were indicated by arrows (not to scale). P: plasmid; C: chromosome; NI: not informed.

PFGE analysis showed that the three MβL-producing *A. baumannii* isolates were not clonally related. Contrastingly, the isolates IEC383 and IEC429 carrying the *bla*_NDM-1_ and *bla*_IMP-1_ genes, respectively, belonged to the same ST (ST464/CC464), while the IEC304 were included in ST216/CC216, as shown in Table. To date, only one NDM-1-producing *A. baumannii* isolate has been reported in Brazil, and it belonged to ST25/CC25.^11^ The other two NDM-1-producing *A. baumannii* strains reported in the continent were both from Colombia, and one of them also belonged to ST464.^15^ ^,^ ^16^ Interestingly, none of the STs associated with NDM-1-producing *A. baumannii* strains described in South America (Fig. 1) belonged to the major clonal complexes CC1, CC15, or CC79, which was responsible for the spread of OXA-23, and more recently, of OXA-72 in *A. baumannii* isolated from this geographic region.^3^ Furthermore, the four MβL-producing *A. nosocomialis* isolates showed distinct PFGE patterns and belonged to CC410 (ST71 and ST1075), CC782 (ST433), and to the singleton ST279 (Table). Rojas and colleagues reported an NDM-1-producing *A. nosocomialis* isolate belonging to ST322 harbouring the same plasmid as that found in an *A. baumannii* strain recovered in Colombia (Fig. 1).^16^ Furthermore, the NDM-1-producing *A. pittii* isolate (IEC37710) reported in the present study was included in ST63/CC63 (Table), differing from the two previous studies that described this MβL in *A. pittii* strains recovered from Brazil (ST119)^5^ and Paraguay (ST320 and ST321).^17^

According to S1 nuclease-PFGE/hybridisation analysis, *bla*_NDM-1_ was located in a ~45-kb plasmid in all six *Acinetobacter* spp. isolates. Previous reports described similar plasmids (~45-55 kb) carrying *bla*_NDM-1_ in distinct *Acinetobacter* species in South America (Fig. 1). This MβL-encoding gene was also reported in a ~100-kb plasmid found in *A. baumannii*,^11^ and in the chromosome of *A. pittii* strains recovered from other Brazilian medical centres.^5^

Initially, the sequencing of a 5,251-bp fragment flanking *bla*_NDM-1_ revealed the presence of the aminoglycoside-modifying enzyme (AME)-encoding gene *aphA6*, followed by IS*Aba125* upstream of *bla*_NDM-1_, which was followed downstream by *ble*_MBL_ (bleomycin resistance) and *ΔtrpF* (phosphoribosylanthranilate isomerase) genes in all NDM-1-producing *Acinetobacter* spp. isolates. This conserved genetic structure (IS*Aba125*-*bla*_NDM-1_-*ble*-*ΔtrpF*) suggests that *bla*_NDM-1_ was inserted in the composite transposon Tn*125*, which has been commonly associated with the mobilisation of this MβL-encoding gene.^5^ ^,^ ^15^ ^,^ ^16^ ^,^ ^17^ ^,^ ^18^ ^,^ ^19^ ^,^ ^20^ To date, Tn*125* has been described among distinct NDM-1-producing *Acinetobacter* species (Fig. 1) recovered from Argentina,^18^ ^,^ ^19^ ^,^ ^20^ Brazil,^5^ ^,^ ^20^ Colombia,^15^ ^,^ ^16^ ^,^ ^20^ and Paraguay.^17^ ^,^ ^20^

**Fig. 2:**
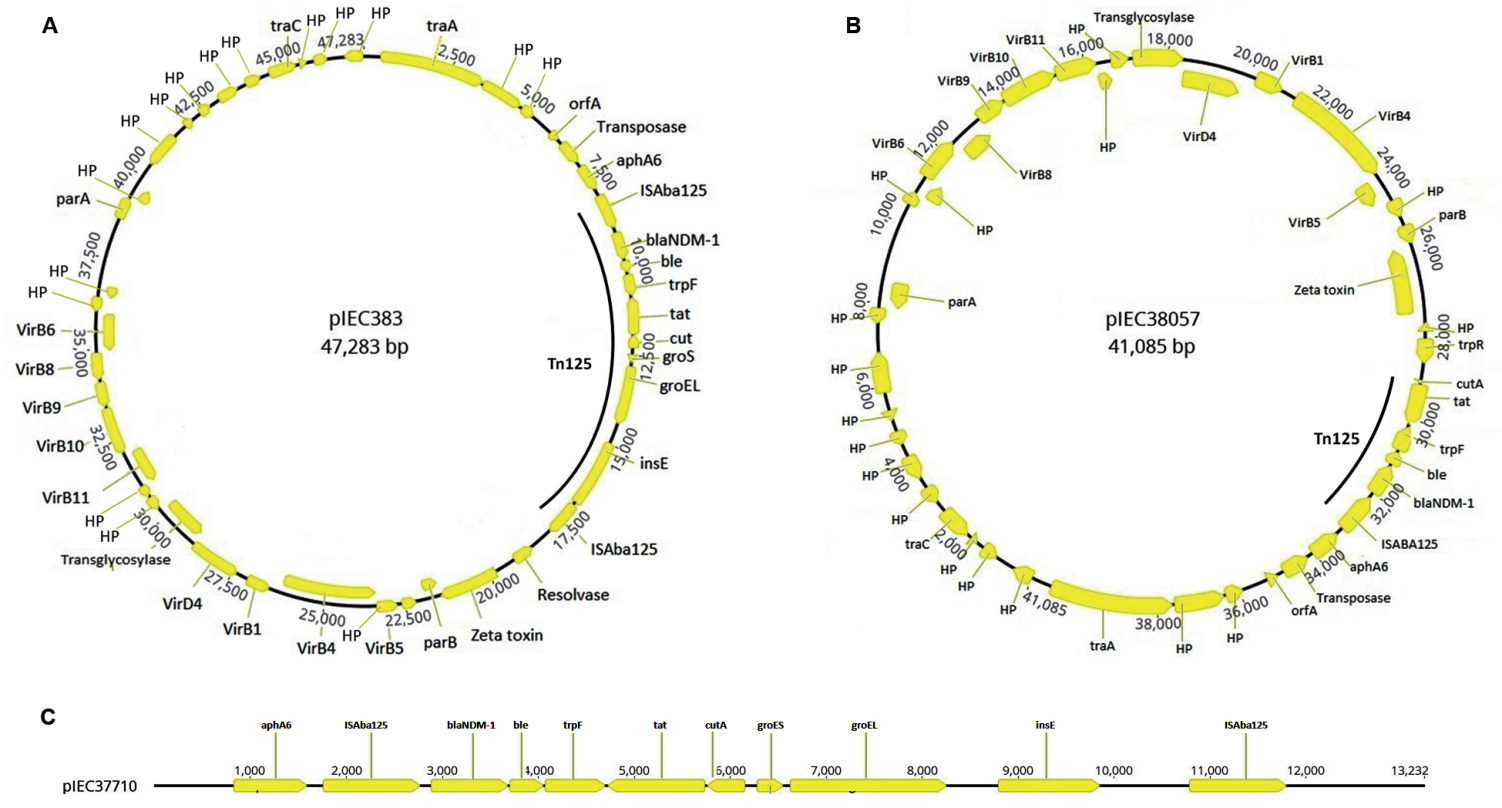
(A) circular map of plasmid pIEC383 recovered from *Acinetobacter baumannii* IEC383. The yellow arrows represent the coding sequences and the direction of transcription. HP: hypothetical protein. The Tn*125* region can be observed. (B) Circular map of plasmid pIEC38057 recovered from *A. nosocomialis* IEC38057. The legend of Fig. 2B was same as that of Fig. 2A. (C) Partial sequence of plasmid pIEC37710 recovered from *A. pittii* IEC37710.

Notably, plasmid sequence analysis revealed that *bla*_NDM-1_ was carried by a 47,283-bp plasmid (pIEC383; Fig. 2A) in the *A. baumannii* IEC383 strain and in a 41,085-bp plasmid (pIEC38057; Fig. 2B) in the *A. nosocomialis* IEC38057 strain that displayed 86% identity at the nucleotide level, corroborating the results obtained by S1 nuclease-PFGE/hybridisation assays. The pIEC383 and pIEC38057 plasmids displayed 95% and 100% identity with pAbNDM-1 and pNDM-BJ01 isolated from *A. baumannii* (JN377410) and *A. lwoffii* (JQ060896) recovered in China, respectively. In addition, both plasmids harboured genes encoding a type IV secretion system that could be related to plasmid conjugation and for a Z toxin of unknown function (Fig. 2A-B). For *A. pittii* IEC37710, only a fragment (13,363 bp; Fig. 2C) of the plasmid pIEC37710 was obtained, displaying 99% identity with pNDM-GJ01 (30,293 bp) isolated from *A. towneri* (KT965092). Interestingly, the three NDM-1-producing *Acinetobacter* species showed distinct Tn*125* backbones (Fig. 1). The Tn*125* structures found in *A. baumannii* IEC383 and in *A. pittii* IEC37710 strains (Fig. 1A,C) were identical to those commonly described among distinct *Acinetobacter* species carrying *bla*_NDM-1_ recovered in Argentina and Colombia (Fig. 1).^15^ ^,^ ^16^ ^,^ ^18^ ^,^ ^20^ However, the comparison between the Tn*125* structures found in *A. pittii* IEC37710 and the other Brazilian *A. pittii* strain carrying *bla*_NDM-1_ in the chromosome was not be possible (Fig. 1), since the Tn*125* had not been fully sequenced.^5^ Moreover, the Tn*125* found in *A. nosocomialis* pIEC38057 showed an unusual structure composed of a truncated *cutA* with a resolvase-encoding gene *tnpR* downstream of *tat*, and the complete absence of *groES-groEL-insE*, as well as the right-side copy of IS*Aba125* (Fig. 1). This deletion of 6,198 bp explains the differences in the plasmid sizes observed between pIEC383 (47,283 bp; Fig. 2A) and pIEC38057 (41,085 bp; Fig. 2B). These results demonstrate a high variability of the Tn*125* genetic backbones among distinct *Acinetobacter* species in South America (Fig. 1), contrasting with the high homology (99-100%) verified among plasmids carrying *bla*_NDM-1_ worldwide.^15^ ^,^ ^16^ ^,^ ^17^

The analysis of the genetic context of *bla*_IMP-1_ showed that it was carried by the class 1 integron In*86* in all *Acinetobacter* spp. isolates. The cassette arrangement structure of In*86* is composed of *bla*_IMP-1_ at the first position, followed by two AME-encoding genes (*aacA31* and *aadA1e*), as previously described.^4^ The In*86* has been described among distinct *Acinetobacter* species, and in other gram-negative bacilli recovered from hospitals in the southeastern Brazil.^4^ Interestingly, the distance between the cities of Belém and São Paulo, where IMP-like-producing *Acinetobacter* spp. strains had been geographically restricted,^4^ ^,^ ^10^ is 2,465 km (1,532 miles).

In conclusion, we reported the spread of NDM-1 and IMP-1 among distinct *Acinetobacter* species in Brazil. The acquisition of MβL-encoding genes by non-*baumannii Acinetobacter* species is of great concern, drastically limiting the therapeutic options for infections caused by such pathogens. In addition, the production of class B carbapenemases seems to be the main mechanism of carbapenem resistance among non-*baumannii Acinetobacter* species in the Brazilian Amazon Region, as well as in other South American countries. The successful dissemination of *bla*_NDM-1_ among different hosts seems to be mainly associated with the plasmid-mediated composite transposon Tn*125* in this geographic region. Therefore, attention should be paid to control the spread of these emerging multidrug-resistant pathogens, particularly among patients hospitalised at ICUs.

## References

[1] World Health Organization (2017). Guidelines for the prevention and control of carbapenem-resistant
enterobacteriaceae, Acinetobacter baumannii and Pseudomonas aeruginosa in
health care facilities. Geneva.

[2] Wong D, Nielsen TB, Bonomo RA, Pantapalangkoor P, Luna B, Spellberg B (2017). Clinical and pathophysiological overview of Acinetobacter
infections a century of challenges. Clin Microbiol Rev.

[3] Vasconcelos AT, Barth AL, Zavascki AP, Gales AC, Levin AS, Lucarevschi BR (2015). The changing epidemiology of Acinetobacter spp producing OXA
carbapenemases causing bloodstream infections in Brazil: a BrasNet
report. Diagn Microbiol Infect Dis.

[4] Cayô R, Streling AP, Nodari CS, Matos AP, de Paula Luz A.Dijkshoorn L (2018). Occurrence of IMP-1 in non-baumannii Acinetobacter clinical
isolates from Brazil. J Med Microbiol.

[5] Pagano M, Poirel L, Martins AF, Rozales FP, Zavascki AP, Barth AL (2015). Emergence of NDM-1-producing Acinetobacter pittii in
Brazil. Int J Antimicrob Agents.

[6] Chagas TP, Carvalho-Assef AP, Aires CAM, Bertocini R, Asensi MD (2015). Detection of an NDM-1-producing Acinetobacter bereziniae strain
in Brazil. J Glob Antimicrob Resist.

[7] ANVISA (Brazilian Health Surveillance Agency) (2018). Boletim segurança do paciente e qualidade em serviços de saúde
nº16: avaliação dos indicadores nacionais das infecções relacionadas à
assistência à saúde (IRAS) e resistência microbiana do ano de
2016. https://www20.anvisa.gov.br/segurancadopaciente/index.php/publicacoes/item/boletim-seguranca-do-paciente-e-qualidade-em-servicos-de-saude-n-16-avaliacao-dos-indicadores-nacionais-das-infeccoes-relacionadas-a-assistencia-a-saude-iras-e-resistencia-microbiana-do-ano-de-2016.

[8] Rodrigues-Costa F, Cayô R, Matos AP, Girardello R, Martins WMBS, Carrara-Marroni FE (2019). Temporal evolution of Acinetobacter baumannii ST107 clone
conversion of bla(OXA-143) into bla(OXA-231) coupled with mobilization of
ISAba1 upstream occAB1. Res Microbiol.

[9] Cardoso JP, Cayô R, Girardello R, Gales AC (2016). Diversity of mechanisms conferring resistance to ß-lactams among
OXA-23-producing Acinetobacter baumannii clones. Diagn Microbiol Infect Dis.

[10] Cayô R, Rodrigues-Costa F, Matos AP, Carvalhaes CG, Jové T, Gales AC (2015). Identification of a new integron harboring bla(IMP-10) in
carbapenem-resistant Acinetobacter baumannii clinical
isolates. Antimicrob Agents Chemother.

[11] Pillonetto M, Arend L, Vespero EC, Pelisson M, Chagas TP, Carvalho-Assef AP (2014). First report of NDM-1-producing Acinetobacter baumannii sequence
type 25 in Brazil. Antimicrob Agents Chemother.

[12] La Scola B, Gundi VA, Khamis A, Raoult D (2006). Sequencing of the rpoB gene and flanking spacers for molecular
identification of Acinetobacter species. J Clin Microbiol.

[13] Vila-Farrés X, Ferrer-Navarro M, Callarisa AE, Martí S, Espinal P, Gupta S (2015). Loss of LPS is involved in the virulence and resistance to
colistin of colistin-resistant Acinetobacter nosocomialis mutants selected
in vitro. J Antimicrob Chemother.

[14] Wang YC, Lee YT, Yang YS, Chen CT, Chiu CH, Yin T (2015). Risk factors and outcome for colistin-resistant Acinetobacter
nosocomialis bacteraemia in patients without previous colistin
exposure. Clin Microbiol Infect.

[15] Marquez-Ortiz RA, Haggerty L, Olarte N, Duarte C, Garza-Ramos U, Silva-Sanchez J (2017). Genomic epidemiology of NDM-1-encoding plasmids in Latin American
clinical isolates reveals insights into the evolution of multidrug
resistance. Genome Biol Evol.

[16] Rojas LJ, Wright MS, De La Cadena E.Motoa G.Hujer KM.Villegas MV (2016). Initial assessment of the molecular epidemiology of blaNDM-1 in
Colombia. Antimicrob Agents Chemother.

[17] Pasteran F, Mora MM, Albornoz E, Faccone D, Franco R, Ortellado J (2014). Emergence of genetically unrelated NDM-1-producing Acinetobacter
pittii strains in Paraguay. J Antimicrob Chemother.

[18] Brovedan M, Marchiaro PM, Morán-Barrio J, Cameranesi M, Cera G, Rinaudo M (2015). Complete sequence of a bla(NDM-1)-harboring plasmid in an
Acinetobacter bereziniae clinical strain isolated in
Argentina. Antimicrob Agents Chemother.

[19] Montaña S, Palombarani S, Carulla M, Kunst A, Rodriguez CH, Nastro M (2017). First case of bacteraemia due to Acinetobacter schindleri
harbouring bla(NDM-1) in an immunocompromised patient. New Microbes New Infect.

[20] Escandón-Vargas K, Reyes S, Gutiérrez S, Villegas MV (2017). The epidemiology of carbapenemases in Latin America and the
Caribbean. Expert Rev Anti Infect Ther.

